# The Late Effects of Radiation Therapy on Skeletal Muscle Morphology and Progenitor Cell Content are Influenced by Diet-Induced Obesity and Exercise Training in Male Mice

**DOI:** 10.1038/s41598-019-43204-8

**Published:** 2019-04-30

**Authors:** Donna D’Souza, Sophia Roubos, Jillian Larkin, Jessica Lloyd, Russell Emmons, Hong Chen, Michael De Lisio

**Affiliations:** 1 School of Human Kinetics, University of Ottawa, Ottawa, ON Canada; 2 Departments of Kinesiology and Community Health, University of Illinois at Urbana-Champaign, Urbana, IL USA; 3 Food Science and Human Nutrition, University of Illinois at Urbana-Champaign, Urbana, IL USA

**Keywords:** Mesenchymal stem cells, Muscle stem cells

## Abstract

Radiation exposure during muscle development induces long-term decrements to skeletal muscle health, which contribute to reduced quality of life in childhood cancer survivors. Whether the effects of radiation on skeletal muscle are influenced by relevant physiological factors, such as obesity and exercise training remains unknown. Using skeletal muscle from our previously published work examining the effects of obesity and exercise training on radiation-exposed bone marrow, we evaluated the influence of these physiological host factors on irradiated skeletal muscle morphology and cellular dynamics. Mice were divided into control and high fat diet groups with or without exercise training. All mice were then exposed to radiation and continued in their intervention group for an additional 4 weeks. Diet-induced obesity resulted in increased muscle fibrosis, while obesity and exercise training both increased muscle adiposity. Exercise training enhanced myofibre cross-sectional area and the number of satellite cells committed to the myogenic lineage. High fat groups demonstrated an increase in p-NFĸB expression, a trend for a decline in *IL-6*, and increase in *TGFB1*. These findings suggest exercise training improves muscle morphology and satellite cell dynamics compared to diet-induced obesity in irradiated muscle, and have implications for exercise interventions in cancer survivors.

## Introduction

Millions of Americans are diagnosed with cancer each year^1^. Fortunately, improvements in the ability to detect and treat cancer has led to 5-year survivor rates following cancer diagnosis of 60%^2^. Approximately two-thirds of these survivors undergo radiation therapy^3^; as such, the late effects of radiation represent an area of clinical concern. Radiation-induced fibrosis is the most common late effects of radiation therapy; affecting up to 80% of irradiated patients^4^. In skeletal muscle, radiation-induced fibrosis result in muscle atrophy, weakness, impaired mobility, increased morbidity and disability, and reduced quality of life^5^. Since a large degree of individual variability exists in both incidence and extent of radiation-induced fibrosis in skeletal muscle, it is important to identify physiological factors that may influence these negative effects. Since, physical inactivity and obesity are associated with a higher risk of several cancers^6^, and a cancer diagnosis is often associated with a reduction in physical activity levels, and increased prevalence of overweight/obesity^7^, comparing how these physiological factors influence radiation-induced atrophy and fibrosis in skeletal muscle could improve clinical outcomes.

Currently, the lack of a complete understanding of the mechanisms responsible for radiation-induced muscle atrophy and fibrosis is a major barrier to identifying therapeutic strategies. Myogenic stem cells, termed satellite cells^8^, contribute to muscle growth^9^ and/or repair^10^. Previous work has shown that irradiated skeletal muscle lacks the capacity to regenerate or respond to hypertrophic stimuli due, at least in part, to radiation-induced satellite cell depletion^11,12^. Satellite cells are regulated, in part, by trophic factors released from non-myogenic, muscle-derived stem/progenitor cells, termed Fibro/Adipogenic Progenitors (FAPs)^13,14^. In healthy skeletal muscles, paracrine factors derived from FAPs promote satellite cell differentiation; however, under pathological conditions, FAP dysregulation can lead to increased fibro/fatty tissue accumulation in skeletal muscle^13,15^. Although the direct effect of radiation on FAPs has not been extensively examined, a model of radiation-induced muscle fibrosis identified that FAPs were localized to areas with increased collagen deposition, and likely contribute to skeletal muscle fibrosis^14^. Collectively, these data suggest that radiation can reduce muscle regenerative capacity and induce fibrosis, partly through adverse effects on muscle-resident stem/progenitor cells. Interestingly, radiation in combination with high fat feeding, further impaired satellite cell function^16^. In contrast, exercise training improved skeletal muscle anti-oxidant capacity, minimizing the harmful effects of radiation exposure^17^. Further, treadmill exercise is known to increase satellite cell^18^ and FAP content in healthy skeletal muscle^19^. As such, these data suggest that obesity may exacerbate the negative effects of radiation on skeletal muscle progenitors, while exercise may be a viable therapeutic option to attenuate these radiation-induced changes.

Thus, the aim of the present study was to compare how diet-induced obesity and exercise training, two modifiable physiological factors relevant to cancer survivors^20^, influence the late effects of radiation exposure on skeletal muscle. Using skeletal muscle from our previously published work examining the effects of obesity and exercise training on radiation-exposed bone marrow^21^, we specifically investigated skeletal muscle morphology and progenitor cell populations, two outcomes known to influence overall muscle health. To achieve this aim, mice were made obese and exposed to exercise training prior to radiation so that radiation would be applied to muscle that is already obese or exercise trained. Obesity and exercise training were continued after radiation exposure to compare how these conditions influence the late effects of therapy as they are developing. To examine late effects, a time point after the acute effects of radiation have subsided was investigated. We hypothesized that compared to obese, male mice, exercise trained, male mice would demonstrate reduce late effects of radiation therapy characterized by improved skeletal muscle morphology and stem/progenitor cell dynamics with reduced fibrosis and adiposity.

## Results

### A high fat diet induces obesity in irradiated mice that is not attenuated with exercise

**Figure 1:**
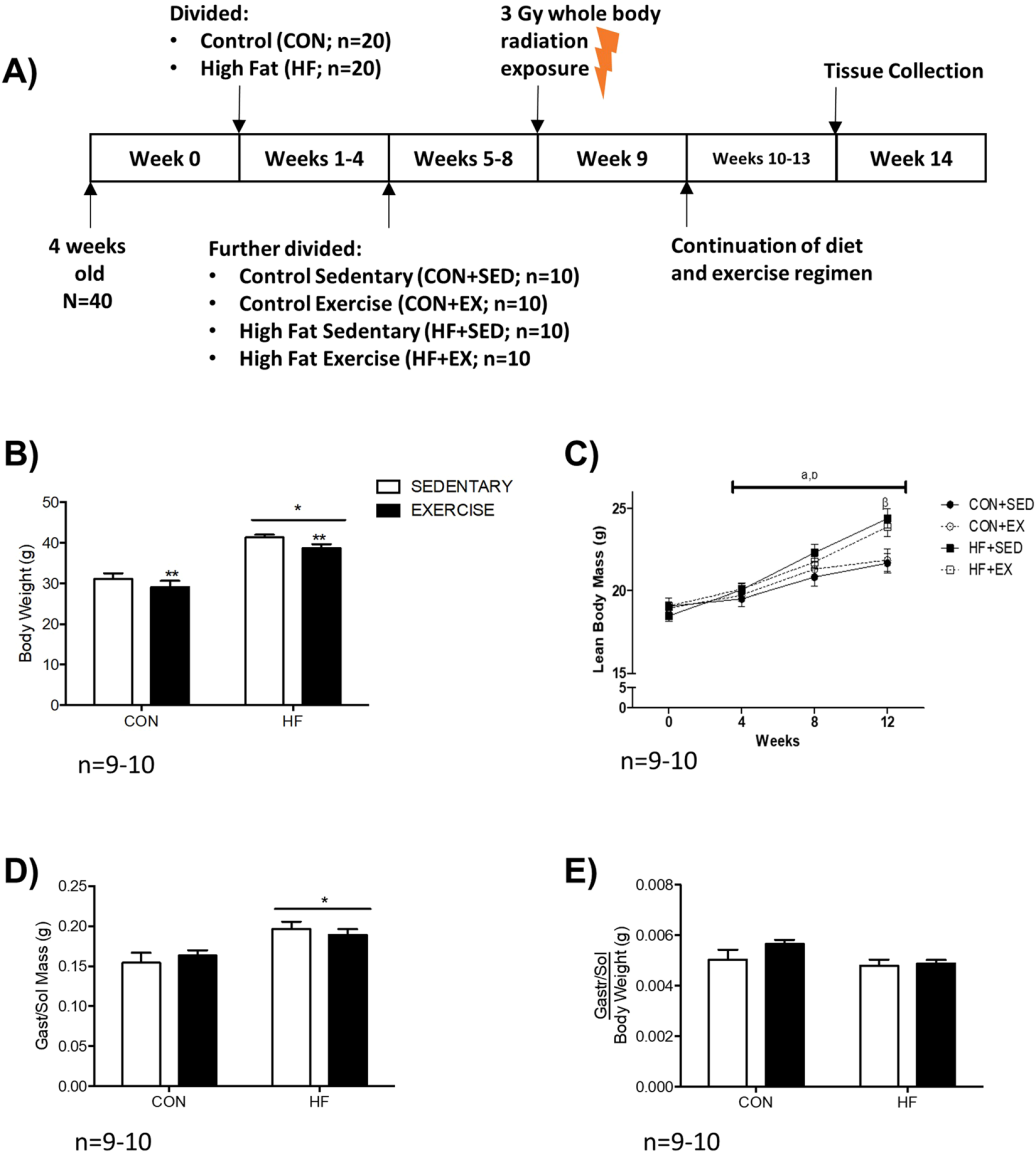
Study Design and Anthropometrics. (**A**) Graphical schematic of study design. (**B**) Body Weight was significantly higher in HF groups compared to CON, with a trend for decreased body weight in EX groups compared to SED. (**C**) After 4 weeks of the diet, HF groups demonstrated a significant increase in lean body mass compared to CON. (**D**) Absolute mass of the gastrocnemius-soleus complex (gastroc-sol). (**E**) No significant difference in gastroc-sol mass was evident after correcting for body weight. *p < 0.05 vs. CON; **p = 0.052 vs. SED. n = 10 CON-SED, n = 9 CON-EX, n = 10 HF-SED, n = 10 HF-EX.

A schematic overview of the present study is shown in Fig. 1A. High fat diet-fed (HF) mice demonstrated a 1.3-fold increase in body weight compared to CON (p < 0.05; Fig. 1B), while a trend for decreased body weight is observed when comparing exercise trained (EX) to sedentary (SED) mice (p = 0.052; Fig. 1B). Lean body mass gradually increased over time, and significantly differed between HF and control diet-fed (CON) groups upon study completion (p < 0.05, Fig. 1C). Compared to CON, HF mice had higher absolute gastrocnemius/soleus complex (gastroc-sol) muscle mass (p < 0.05, Fig. 1D), but these differences were not observed when normalized to body weight (Fig. 1E).

### In exercise trained mice exposed to radiation, myofibre size is increased independent of diet, while increased myonuclear content changes are diet-dependent

**Figure 2:**
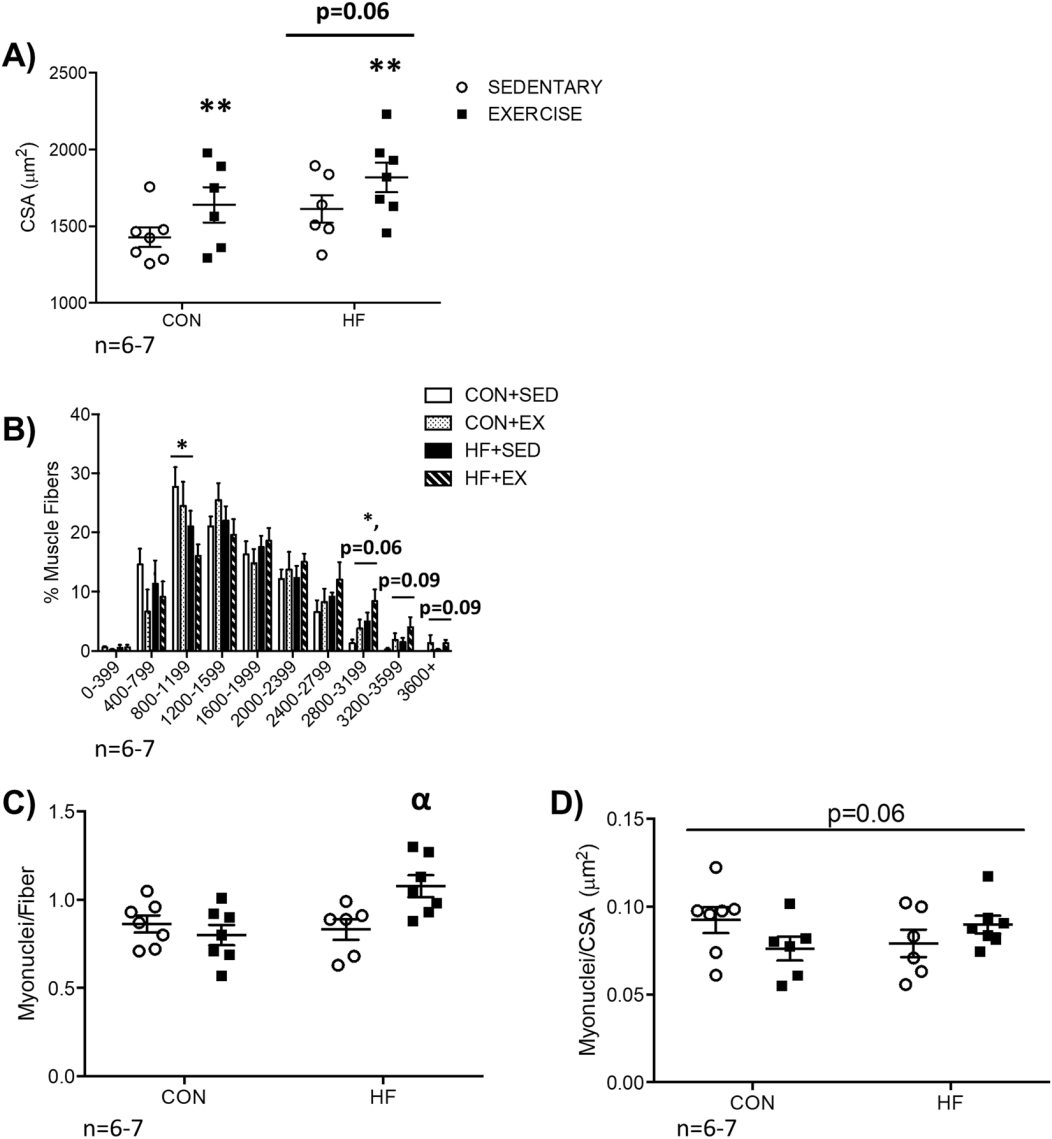
Skeletal Muscle Morphology. (**A**) Exercise trained groups demonstrated a significant increase in gastroc-sol cross-sectional area (CSA). (**B**) An overall shift in myofibre distribution is observed following diet and exercise. Specifically, a decrease in the frequency of smaller-sized myofibres is observed in HF mice compared to CON, while an increase in the number of larger fibres was identified in HF (compared to CON) and EX (compared to SED, p = 0.06 and p = 0.09). (**C**) Myonuclei per fibre was significantly increased in HF-EX, compared to HF-SED with no difference between CON-EX and CON-SED. (**D**) A trend for an interaction effect was identified in myonuclear domain (myonuclei/CSA, p = 0.06). **p < 0.05 vs. SED, *p < 0.05 vs. CON, ^α^p < 0.05 vs. HF-SED. n = 6–7.

In EX mice exposed to radiation myofibre cross-sectional area was increased (CSA; Fig. 2A, p < 0.05), while a trend for an increase was observed in obese mice (Fig. 2A, p = 0.06). In HF mice exposed to radiation, the frequency of 800–1199 μm^2^ fibres was lower compared to CON, while the frequency of 2800–3199 μm^2^ fibres was higher (Fig. 2B, both p < 0.05). In EX mice exposed to radiation, there was a trend for more 2800–3199 μm^2^ fibres (p = 0.06), 3200–3599 μm^2^ fibres (p = 0.09), and 3600^+^ μm^2^ fibres (p = 0.09) compared to SED (Fig. 2B). In HF-EX mice exposed to radiation, myonuclei content was higher compared to HF-SED with no difference between CON-SED and CON-EX mice exposed to radiation (Fig. 2C, p < 0.05). A trend for an interaction effect between HF and EX mice exposed to radiation was observed for myonuclear domain size, defined as the number of myonuclei per CSA (p = 0.06, Fig. 2D).

### In obese mice exposed to radiation, muscle fibrosis is elevated, while both obese and exercise trained mice exposed to radiation have increased muscle adiposity

**Figure 3:**
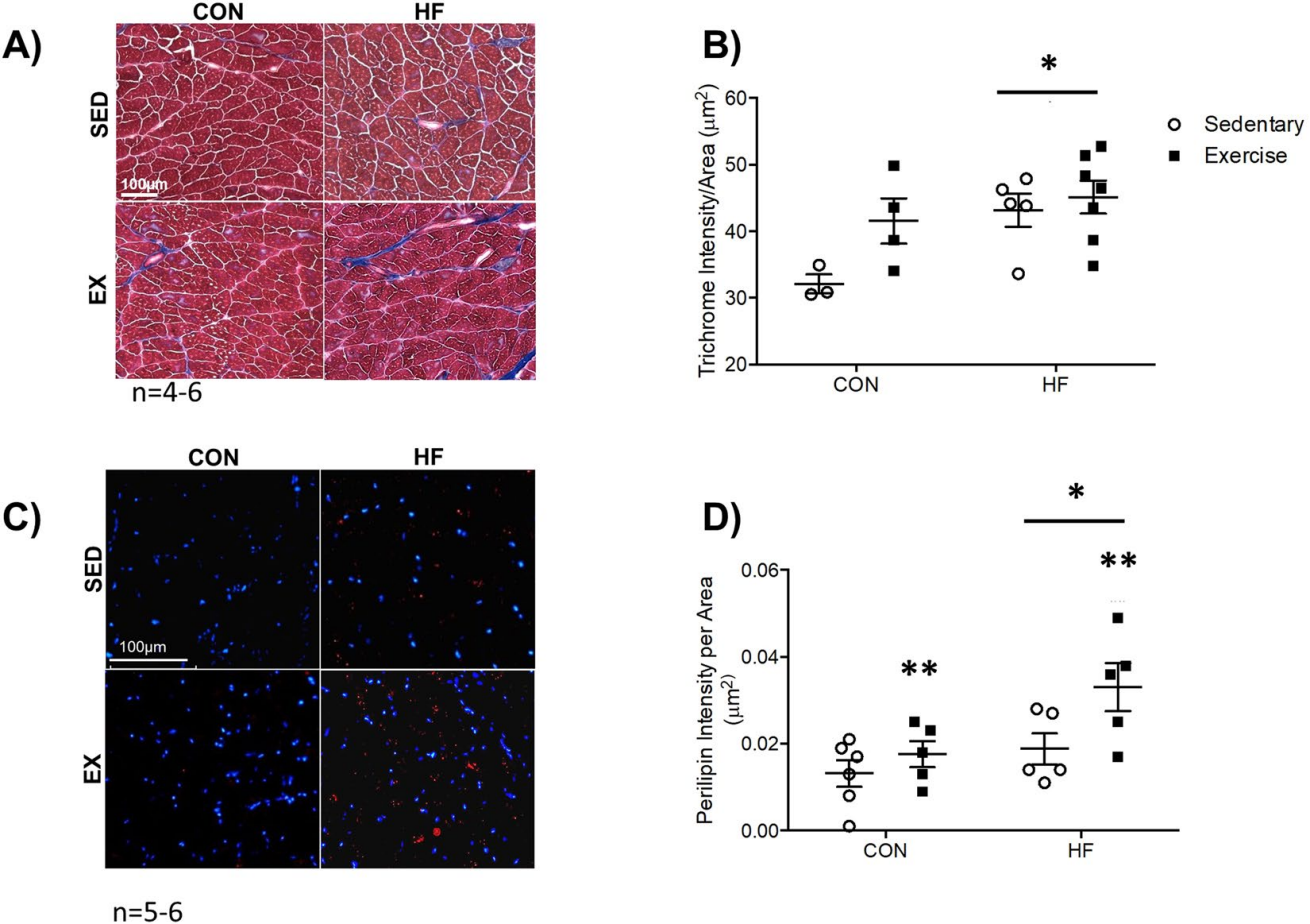
Skeletal Muscle Fibrosis and Adiposity. (**A**,**B**) A representative image and corresponding graph of Trichrome staining demonstrates that HF feeding is associated with an increase in muscle fibrosis. n = 4–6. (**C**,**D**) A representative image and corresponding graph of Perilipin immunofluorescence reveals that diet and exercise independently enhanced skeletal muscle adiposity. n = 5–6. *p < 0.05 vs. CON, **p < 0.05 vs. SED.

Representative Trichrome images are presented in Fig. 3A. In HF mice exposed to radiation, trichrome stain intensity was significantly elevated compared to CON (Fig. 3B, p < 0.05). In both HF and EX mice exposed to radiation, Perilipin staining intensity (Fig. 3C) revealed that muscle adiposity was significantly higher compared to CON and SED (Fig. 3D, both p < 0.05).

### Exercise training increases skeletal muscle satellite cell myogenic commitment, irrespective of high fat feeding in irradiated mice

**Figure 4:**
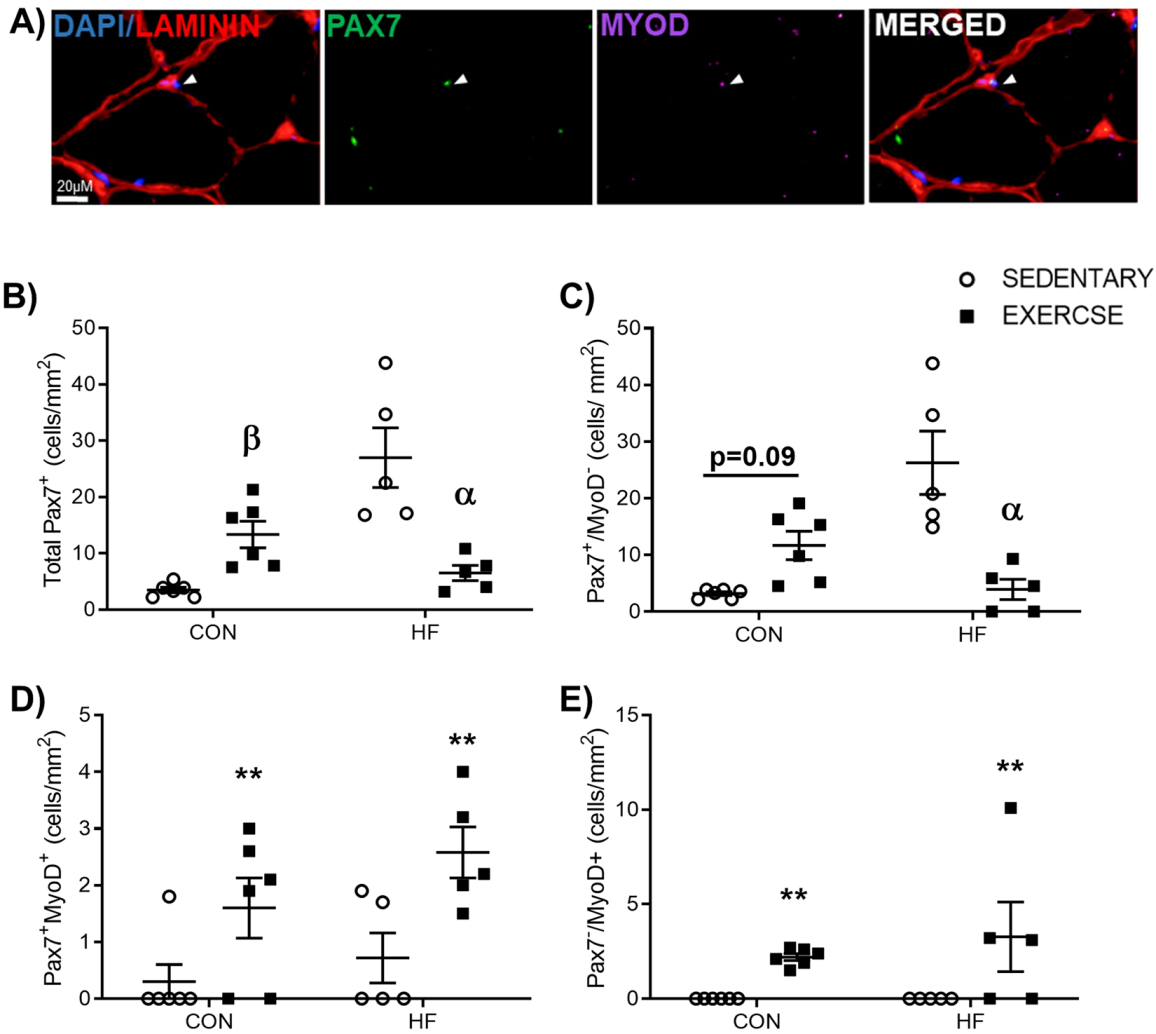
Satellite Cell Dynamics. (**A**) Representative image of immunofluorescent staining for satellite cell (SC) markers. (**B**) Total SC content (Total Pax7^+^) and (**C**) quiescent SC content (Pax7^+^/MyoD^−^) were influenced by a combination of exercise, dependent on diet condition. A significant increase in total SC content was observed in CON-EX versus CON-SED, while a significant decrease in total SC content was observed in HF-EX versus HF-SED (**B**). Similarly, a trend for an increase in quiescent SC content was observed in CON-EX versus CON-SED (p = 0.09), while a significant decrease in quiescent SC content was observed in HF-EX versus HF-SED. Exercise facilitated an increase in SC myogenic commitment, regardless of diet, as identified through quantification of the number of (**D**) Pax7^+^/MyoD^+^ and (**E**) Pax7^−^/MyoD^+^ SCs. ^β^p < 0.05 vs. CON-SED, ^α^p < 0.05 vs. HF-SED, and **p < 0.05 vs. SED. n = 5–6.

Pax7/MyoD co-staining was conducted to evaluate satellite cell population dynamics^22^ (Fig. 4A). A significant interaction effect was detected for total Pax7^+^ cell content (Fig. 4B). Total Pax7^+^ cells were significantly higher in CON-EX versus CON-SED (Fig. 4B, p < 0.05), and significantly lower in HF-EX compared to HF-SED (Fig. 4B, p < 0.05). Similarly, a significant interaction was detected in Pax7^+^/MyoD^−^ quiescent satellite cells (Fig. 4C). Pax7^+^/MyoD^−^ cells trended higher in CON-EX versus CON-SED (p = 0.09), and was significantly lower in HF-EX versus HF-SED (p < 0.05). The number of committed Pax7^+^/MyoD^+^ and differentiated Pax7^−^/MyoD^+^ satellite cells was significantly higher in exercise trained groups compared to sedentary groups independent of diet (Fig. 4D,E, both p < 0.05).

### Exercise training differentially modulates FAP content in control diet and high fat diet-fed, irradiated muscle

**Figure 5:**
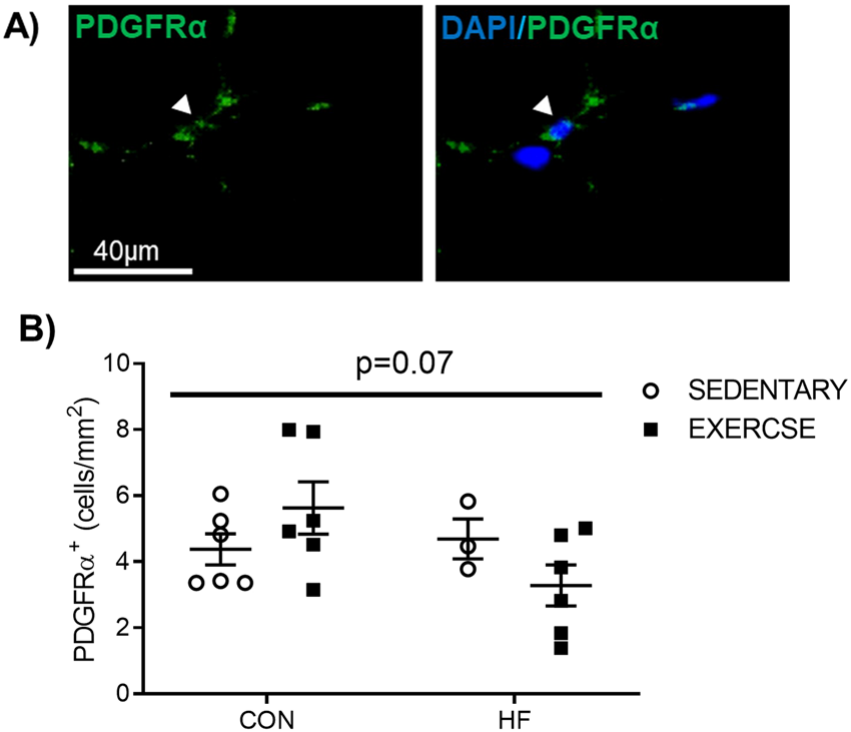
Fibro/Adipogenic Progenitor (FAP) Content. (**A**) Representative image and (**B**) graphical presentation of Fibro/Adipogenic Progenitor (FAP) cell quantification. FAP cells were evaluated by counting the number of PDGFRα+ cells through immunofluorescent staining. A trend for higher FAP content in CON-EX and a trend for lower FAP content in HF-EX, irradiated muscle was observe (interaction effect, p = 0.07). n = 3–6.

FAP content was determined through quantification of PDGFRα+^13^ interstitial cells in all experimental groups (Fig. 5A). A trend for an interaction in FAP content between high fat feeding and exercise was observed with exercise training increasing FAP content in control diet-fed mice and reducing FAP content in high fat diet-fed mice (Fig. 5B, p = 0.07).

### High fat feeding in irradiated muscle enhances p-NFĸB content, and somewhat influences inflammatory and fibrotic gene expression

**Figure 6:**
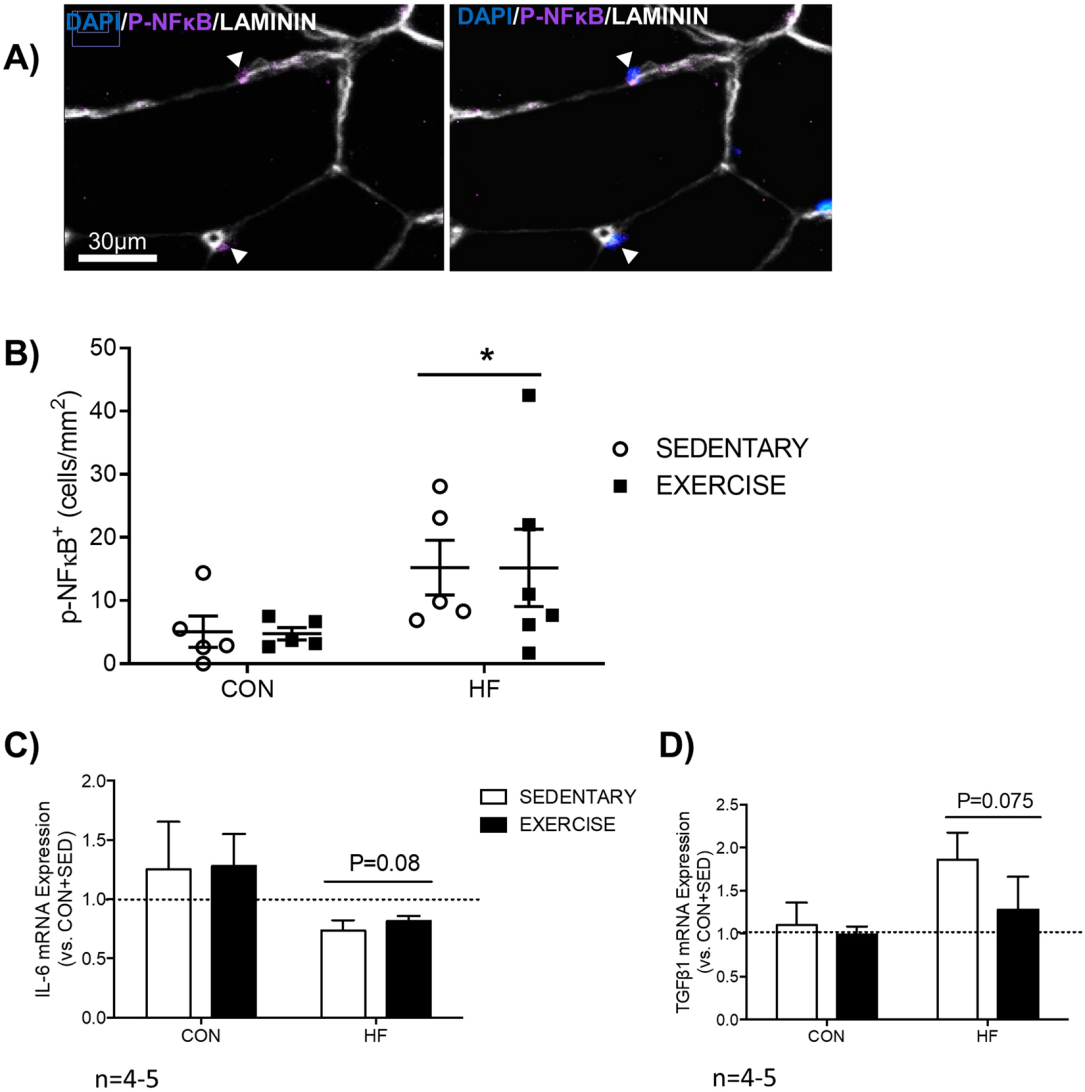
Skeletal Muscle Inflammation. (**A**) Representative and (**B**) graphical presentation of quantification of phosphorylated NFκB (P-NFκB), an established marker of inflammation, demonstrates that high fat feeding had a marked effect on skeletal muscle inflammation, irrespective of exercise training. *p < 0.05 vs. CON, n = 5–6. Genes associated with FAP paracrine secretion (**C**) and fibrosis (**D**) were examined through qPCR of skeletal muscle. All values are normalized to β2M (β2 microglobulin) and are expressed relative to CON + SED. n = 4–5.

Representative immunofluorescence images of p-NFĸB nuclei are presented (Fig. 6A). High fat fed mice had significantly higher p-NFĸB^+^ nuclei (Fig. 6B, p < 0.05). High fat feeding facilitated a trend for a decrease in *IL-6* (P = 0.08, Fig. 6C), an established FAP paracrine factor^15,23^, and a trend for an increase in *TGFβ1* (P = 0.075, Fig. 6D), a key contributor to skeletal muscle fibrosis^24^. Gene expression of macrophage markers *F4/80* and *CD206*, inflammatory cytokine *TNFα*, and *Type 1 Procollagen (Col1a*) was not significantly affected by high fat feeding and exercise (Supplementary Fig. 1A–D).

## Discussion

The aim of the current study was to investigate the effects of two modifiable lifestyle factors, diet and exercise, on morphology and stem/progenitor cell content in irradiated skeletal muscle. Our results suggest that high fat feeding adversely affects skeletal muscle adiposity and fibrosis, independent of exercise training, while exercise training with or without high fat feeding, improved satellite cell population dynamics and increased myofibre size in irradiated muscle. These findings have implications for dietary and lifestyle recommendations to maintain health of irradiated skeletal muscle in cancer survivors.

Myofibre CSA and fibro/fatty tissue accumulation are two key aspects of muscle health that relate to force production and glucose metabolism. Previous work has demonstrated that the effects of high fat feeding or exercise training alone on developing myofibre CSA may depend on fibre type with changes in response to both interventions more prevalent in type I compared to type II fibres^25,26^. When high fat diet and exercise are combined, a previous study indicated that myofibre CSA of the gastrocnemius/soleus complex (comprised of both fibre types) did not change^27^. Contrary to these results, we observed a significant increase in average myofibre CSA of gastrocnemius/soleus muscle complex, regardless of diet. Similar to previous studies^27,28^, we observed an increase in fibro/fatty tissue accumulation in high fat diet-fed mice; however, this was not attenuated by exercise, which conflicts with previous work^27^. Conflicting results for myofibre CSA and muscle fibrosis are likely due to differences in the duration and speed of exercise training, proportion of fat in the diet, and/or radiation exposure. Additionally, while previous work introduced high fat feeding and exercise simultaneously^27^, the present study initiated high fat feeding 4 weeks prior to beginning the exercise protocol, and previous work has shown that high fat feeding initiates muscle fibrosis^28^ that cannot be reversed with exercise.

Unlike fibrosis, changes to skeletal muscle adiposity observed in the current study align with previous literature. The significant increase in Perilipin, an established contributor to lipid storage^29^, identified in HF-EX mice coincides with prior findings that demonstrate augmented adiposity following high fat feeding^30^ and endurance training^31^. Both intramyocellular lipids and intermuscular adipose tissue increase in obesity and are negatively related to insulin sensitivity^32^. Paradoxically, exercise training is also known to increase intramyocellular lipids despite improving insulin sensitivity^33^. It has been suggested that the proximity of intramyocellular lipids and mitochondria explain the apparent contradiction between obese and trained skeletal muscle^34^. Due to the nature of the analysis, we were unable to determine if the location of lipid accumulation differed between high fat fed and exercise trained skeletal muscle. Future work should more closely examine the distribution of intramuscular lipids throughout irradiated skeletal muscle following high fat feeding and/or exercise training. Together, these findings suggest that in irradiated muscle, exercise training can partially inhibit some of the negative morphological changes induced by a high fat diet.

To examine the potential cellular contributors to the observed morphological differences, we investigated the dynamics of muscle-resident stem/progenitor cells. In the current study, exercise training differentially influenced total and quiescent satellite cell content in a diet-dependent manner. Exercise training in control diet conditions increased total satellite cells and trended to increase quiescent satellite cells (Pax7^+^/MyoD^−^) in irradiated mice. Conversely, exercise training in high fat diet-fed mice reduced total and quiescent satellite cell content. Regardless of diet, exercise training increased SC commitment to the myogenic lineage, indicated by an increase in Pax7^+^/MyoD^+^ and Pax7^−^/MyoD^+^ satellite cells. These findings align with our observation that the highest myonuclear content, a marker of satellite cell fusion, was observed in HF-EX mice. Further, myonuclear domain trended to be reduced in HF-SED compared to CON-SED, and increased in HF-EX compared to CON-EX, suggesting impaired myogenic commitment in HF-SED mice. Indeed, impaired satellite cell commitment to the myogenic lineage has been identified using various rodent models of obesity^35–37^, while exercise training, irrespective of diet, has been found to enhance myogenic commitment^38^. Thus, we speculate that high fat feeding may prevent myogenic commitment of satellite cells, while exercise training likely provides a stimulus that supports myogenesis to increase myonuclear content in irradiated skeletal muscle.

The precise mechanisms mediating the opposing influence of high fat feeding and exercise on satellite cell function remain poorly understood. FAPs are PDGFRα^+^ cells that extensively proliferate following muscle injury to facilitate muscle repair^13^, and promote myogenic differentiation through paracrine mechanisms^15,39^. Conversely, under pathological conditions, FAPs are responsible for fibro/fatty tissue accumulation in skeletal muscle^13,15^. In the current study, opposing trends for higher FAP content in CON-EX versus CON-SED and lower FAP content in HF-EX versus HF-SED were observed, suggesting diet-dependent effects of exercise on FAPs in irradiated skeletal muscle. It is interesting to speculate that exercise alters the recently identified FAP subpopulations^40^ in a pathology-dependent manner, with an increase in regenerative FAPs in control diet-fed muscle. Supporting this speculation, we observed a trend for a reduction in *IL-6* gene expression in HF groups. This may be related to unfavourable changes to FAP function, given that IL-6 is an established paracrine factor released from FAPs following muscle injury^15,41^. Further investigation is warranted as no study to date has evaluated changes to FAP function and subpopulations following a combination of high fat diet and exercise.

In addition to FAPs, skeletal muscle inflammation represents another key variable that could influence the satellite cell milieu, and is associated with both obesity^42^ and radiation exposure (for review see^43^). Chronic inflammation negatively influences extracellular matrix remodeling by facilitating an increase in fibrosis^44^, which subsequently impairs satellite cell function^45^. Obesity is associated with chronic inflammation^42^, while exercise has been found to reduce inflammation^46^. Increased expression of the inflammatory marker p-NFĸB was observed in both HF groups, regardless of exercise training, and coincides with a trend for an increase in the fibrotic regulator, *TGFβ1* in high fat diet-fed mice. These data suggest that in irradiated skeletal muscle, high fat feeding promotes an inflammatory environment that cannot be fully restored by exercise.

Some limitations of our study should be noted. The present study analyzed cellular dynamics in skeletal muscle at a single time point after radiation, when the acute effects are expected to have resolved. This likely contributed to the lack of difference in inflammatory factor gene expression, but permitted a more accurate depiction of the late effects of therapy. The single time point post-radiation also did not allow us to determine if any differences were present in our groups prior to radiation exposure which would have required a pre-radiation collection for comparison. Further investigation into the time course of skeletal muscle changes following radiation exposure is warranted. Further, the study was not performed in mice with cancer. Including an active tumor in the present model would have introduced known confounding effects of the tumor on muscle health^47^. While this would have increase the clinical translatability of the model, it would have introduced another variable to consider when interpreting the findings. Lastly, obesity and exercise training were induced prior to radiation exposure.

The current study is the first investigation that examines the effects of common lifestyle factors (diet and exercise) in irradiated skeletal muscle. The morphological changes observed demonstrate high fat feeding primarily alters the non-myocellular components of irradiated skeletal muscle, while exercise training primarily impacts myofibre morphology and satellite cell population dynamics. The negative impact of a high fat diet on muscle morphology and the positive impact of exercise on stem/progenitor cell populations should be considered when implementing interventions in irradiated cancer survivors to better preserve muscle health.

## Materials and Methods

### Mice

Four-week-old male CBA (Jackson Laboratories) mice were received and maintained on a 12:12 h light-dark schedule with food and water provided *ad libitum*. Mice acclimated to the facility during Week 0 of the study. At the start of Week 1, mice were randomly assigned to Control (CON; AIN-93M Research Diets, n = 20) or 45% High Fat (HF; D12451 Research Diets, n = 20) diet groups. This study represents analysis of skeletal muscle from mice in a separate manuscript from our group^21^. Ethical approval was obtained from the Illinois Institutional Animal Care and Use Committee, and all experiments were performed in accordance with relevant guidelines and regulations.

### Exercise protocol

At the start of Week 5 (9 weeks of age), mice from CON and HF groups were further randomly divided into exercise or non-exercise conditions to create 4 experimental groups (Control Sedentary, CON + SED; Control Exercise, CON + EX; High Fat Sedentary, HF + SED; High Fat Exercise, HF + EX). Each experimental group contained 10 mice.

Mice were exercised 3 days per week (M/W/F), at the same time each day. Warm up and cool down consisted of running for 5 minutes at 8 m/min. Each training period progressed from 10 m/min for 25 minutes to 23 m/min for 45 minutes. The duration of each training period was between 25 to 45 minutes in length. Mice were motivated to exercise using bristles of a paintbrush. Non-exercised mice were exposed to comparable treadmill stress conditions.

### Total body irradiation

At Week 9 (13 weeks of age), all mice were exposed to a 3 Gy dose of whole body gamma-radiation, as previously described^21^. To prevent movement during radiation administration, mice were anesthetized with an intraperitoneal injection of ketamine/xylazine (65/4 mg/kg). A Theratron 780 Cobalt 60 radiation unit applied uniform dose distribution at a fixed rate of 37.7 cGy/min. Mice were evaluated daily following radiation, and were provided Jell-O to ensure sufficient calorie intake during recovery.

### Tissue collection

Mice were euthanized by CO_2_ inhalation followed by cervical dislocation. Gastrocnemius-Soleus (gastroc-sol) complexes were excised, and either covered in optimum cutting temperature embedding compound prior to freezing in isopentane cooled by liquid nitrogen, or flash frozen and weighed.

### Histochemical and immunofluorescent analyses

Muscles were cut into 10 μm muscle cross-sections and mounted on glass slides to be stained, as described below. Trichrome staining was conducted by the Histology Core Facility at the University of Ottawa following Masson’s Trichrome protocol^48^. For immunofluorescence analyses, muscle cross-sections were fixed with 2% paraformaldehyde (PFA) for 7 minutes, permeabilized using 0.1% Triton X-100 (Thermo Fisher Scientific, Waltham, MA) for 10 minutes at room temperature, blocked using either the M.O.M Blocking kit (Pax7; Vector Labs, Burlingame, CA), DAKO Serum-Free Protein Block (PDGFRα; Agilent, Santa Clara, CA), or standard serum block (Laminin, Perilipin, and p-NFkB; PBS with 5% NGS, 1% BSA, and 0.1% Triton X-100). Sections were incubated with primary antibody overnight at 4 °C (mouse anti-Pax7, DSHB, neat; rabbit anti-MyoD, Abcam, 1:200; rat anti-Laminin, ThermoFisher Scientific, 1:200; rabbit anti-PDGFRα, R&D Systems; 1:250; rabbit anti-phospho-NFĸB, Cell Signaling, 1:200; Perilipin, Cell Signaling, 1:100). Appropriate Alexa secondary antibodies (Invitrogen, Carlsbad, CA) were used for detection of each primary antibody (1:300), while nuclei were counterstained using 4,6-diamidino-2-phenylindole (DAPI, 1:10000).

### Image analysis

Images were acquired using an EVOS-FL2 Automated Microscope (ThermoFisher Scientific; Waltham, MA). Images were scanned at 20x magnification and analyzed using Fiji software^49^. Measurement of muscle cross-sectional area (CSA) was achieved by circling at least 150 myofibres per muscle section. Myonuclei content was determined by counting all myonuclei within each myofibre, with greater than 50% of the nuclei below the basal lamina. Myonuclear domain was established by dividing the myonuclear content of each myofibre analyzed by its respective cross-sectional area. Adiposity and fibrosis were quantified by thresholding each image so that the component interest in each section (i.e. Perilipin or Collagen) was accurately identified by the software. Three to five Regions of Interest (ROIs) were created in random areas of each gastroc-sol section. These ROIs were created to avoid areas that would confound analyses (tears in the section, section folds, areas of freeze damage). The Integrated Density of each ROI was determined, and subsequently divided by its area (μm^2^). An average of these values (Integrated Density of ROI/Area of ROI) was obtained for each section. Stem cell populations and p-NFĸB were quantified by counting the number of positive signals per ROI area (μm^2^).

### Real-Time quantitative PCR

Approximately 30–60 mg of tissue was homogenized in 1 mL Trizol using a Bead Mill Homogenizer (Fischer Scientific, Hampton, NH). Extracted RNA was precipitated overnight at −20 °C, and subsequently pelleted, dried, and re-suspended in 20 μl of sterile water, as previously described^50^. Total RNA (1 µg) was reversed transcribed into cDNA using the High Capacity cDNA Reverse Transcription Kit (Applied Biosystems, Foster City, MA). Quantitative PCR was performed using the BioRad CFX386 QPCR System (Hercules, CA) with Taqman Universal PCR Master Mix (Applied Biosystems). β2 microglobulin (β2M) was used as the housekeeping gene, and all data are represented relative to its expression using the ΔΔCq method^51^. CON + SED was used as a reference group for relative gene expression. Taqman primers for β2M, Emr-1 (F4/80), macrophage mannose receptor 1 (MRC-1/CD206), tumor necrosis factor α (TNFα), interleukin 6 (IL-6), transforming growth factor β1 (TGFβ1), and collagen 1α (Col1α) were all purchased from Applied Biosystems. Gene expression assay ID numbers are provided in Supplementary Table 1.

### Statistical analysis

A two-way ANOVA with Sidak post-hoc analysis (for non-repeated measures) or Tukeys’ post-hoc test (for repeated measures) was performed to determine differences between all four experimental groups. When a significant interaction was detected, comparisons were made between SED and EX within each diet condition. Body weight and changes in lean body mass were analyzed via a repeated measures two-factor ANOVA. Data are reported as a mean + standard error of the mean, with P < 0.05 considered significant.

## Supplementary information

Supplementary Figure 1

## Data Availability

The authors will make all raw data, materials, and protocols freely available upon request.
