# Supplementary material for: The Late Effects of Radiation Therapy on Skeletal Muscle Morphology and Progenitor Cell Content are Influenced by Diet-Induced Obesity and Exercise Training in Male Mice
Source: Sci Rep. 2019 Apr 30;9:6691. doi: 10.1038/s41598-019-43204-8 (PMC6491594; doi:10.1038/s41598-019-43204-8)
Supplement: Supplementary file 1 — Supplementary Figure 1 [file 41598_2019_43204_MOESM1_ESM.pdf]

The Late Effects of Radiation Therapy on Skeletal Muscle Morphology and Progenitor Cell Content are Influenced by Diet-Induced Obesity and Exercise Training in Male Mice

Donna D'Souza[1], Sophia Roubos[1], Jillian Larkin[1], Jessica Lloyd[1], Russell Emmons[2], Hong Chen[3], and Michael De Lisio[1]

[1]School of Human Kinetics, University of Ottawa, Ottawa, ON. [2]Departments of Kinesiology and Community Health, and [3]Food Science and Human Nutrition, University of Illinois at Urbana-Champaign, Urbana, IL.

SUPPLEMENTARY INFORMATION

Supplementary Table 1. Gene expression assay ID numbers.

Gene	***Assay ID***
emr1	Mm00802529_m1
MRC1	Mm00485148_m1
interleukin 6	Mm00446190_m1
TNF α	Mm00443258_m1
β2 microglobulin	Mm00437762_m1
Tgfβ1	Mm01178820_m1
collagen1 α1	Mm00801666_g1

SUPPLEMENTARY FIGURE 1

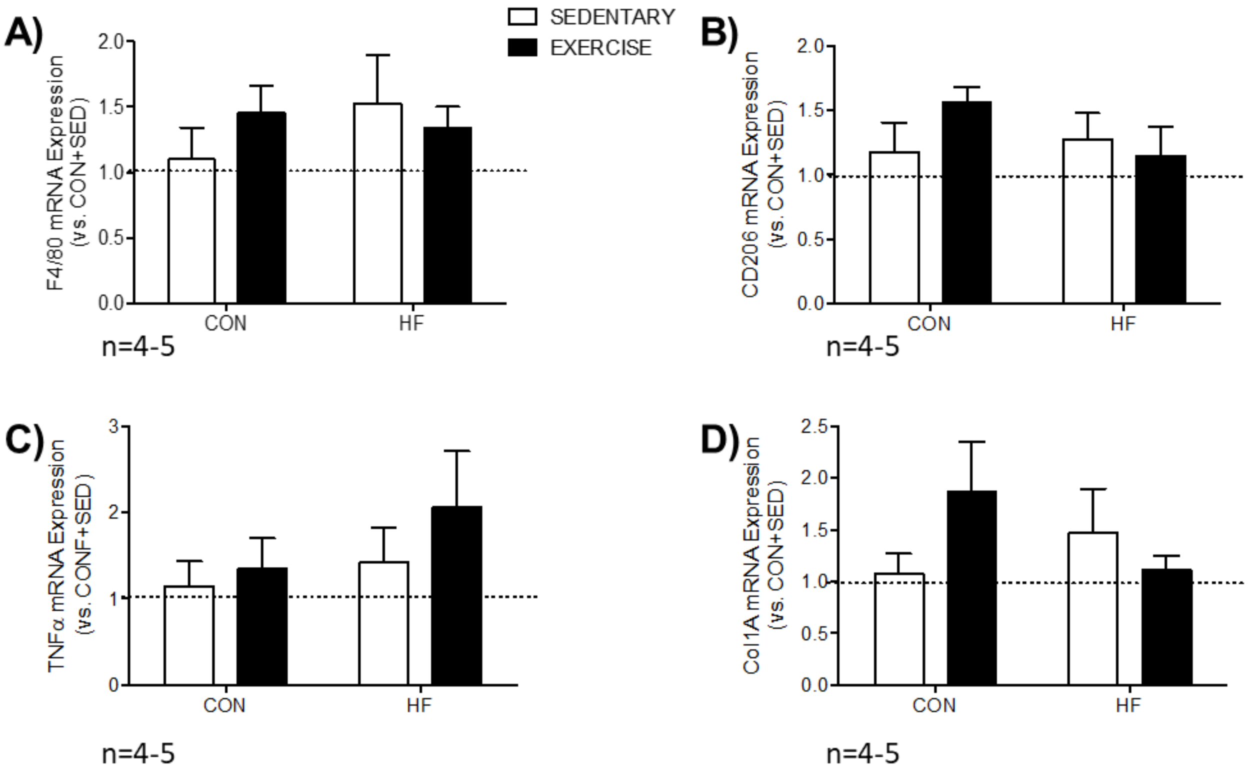

SUPPLEMENTARY FIGURE 1. Inflammatory and Fibrotic Gene Expression

Genes associated with macrophage content (A&B), inflammation (C), and collagen accumulation (D) were examined using qPCR in skeletal muscle. All values are normalized to β2M (β2 microglobulin) and are expressed relative to CON+SED. n=4-5.
